# The Contribution of the Immune System in Bone Metastasis Pathogenesis

**DOI:** 10.3390/ijms20040999

**Published:** 2019-02-25

**Authors:** Lisha Xiang, Daniele M. Gilkes

**Affiliations:** 1 State Key Laboratory of Biotherapy and Cancer Center, Sichuan University, Chengdu 610041, China; lishaxiang@foxmail.com; 2 Breast & Ovarian Cancer Program, Department of Oncology, The Johns Hopkins University School of Medicine, The Sidney Kimmel Comprehensive Cancer Center, Baltimore, MD 21231, USA; 3 Department of Chemical and Biomolecular Engineering, The Johns Hopkins University, Baltimore, MD 21218, USA

**Keywords:** bone metastasis, immune system, immunotherapy

## Abstract

Bone metastasis is associated with significant morbidity for cancer patients and results in a reduced quality of life. The bone marrow is a fertile soil containing a complex composition of immune cells that may actually provide an immune-privileged niche for disseminated tumor cells to colonize and proliferate. In this unique immune milieu, multiple immune cells including T cells, natural killer cells, macrophages, dendritic cells, myeloid-derived suppressor cells, and neutrophils are involved in the process of bone metastasis. In this review, we will discuss the crosstalk between immune cells in bone microenvironment and their involvement with cancer cell metastasis to the bone. Furthermore, we will highlight the anti-tumoral and pro-tumoral function of each immune cell type that contributes to bone metastasis. We will end with a discussion of current therapeutic strategies aimed at sensitizing immune cells.

## 1. Introduction

Accompanied by an increase in the incidence of cancer over the past several decades, bone metastasis has become an ongoing clinical problem which is a major cause of mortality for thousands of patients suffering from cancer. Over 80% of patients with advanced breast cancer or prostate cancer develop bone metastasis, followed by patients with thyroid cancer (60%), lung cancer (30–40%), and renal cancer (20–25%) [1]. Although there have been advances in the diagnosis and treatment of cancer, bone metastasis is still incurable. 

**Figure 1:**
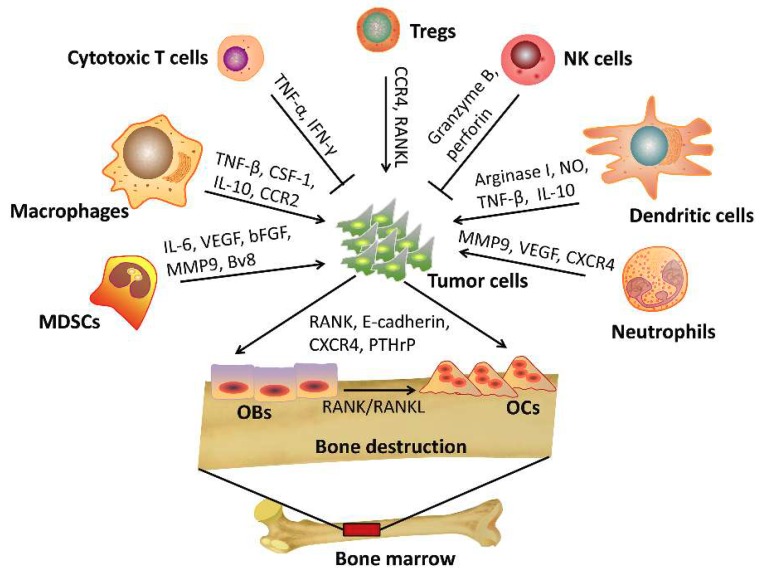
The interaction of immune cells and cancer cells during bone metastasis. Cytotoxic CD8^+^ T cells release TNF-α and IFN-γ to eliminate tumor cells. Natural killer cells (NK cells) kill tumor cells through granzyme B- and perforin-mediated apoptosis. Regulatory T cells (Tregs) promote tumor cell to bone metastasis through CXCR4/CXCL12 signaling or RANK/RANKL axis. Tumor-associated macrophages (TAMs) promote tumor cell to bone metastasis through CCL2/CCR2 or CSF-1/ CSF-1R signaling. Meanwhile, TAMs secret high levels of IL-10 and TGF-β to decrease the activation of CD4^+^ and CD8^+^ T cells. Dendritic cells (DCs) suppress the cytotoxic capacity of CD8^+^ T cells via production of arginase I, nitric oxide (NO), TGF-β, interleukin-10 (IL-10) to promote tumor progression. Myeloid-derived suppressor cells (MDSCs) release chemokines including IL-6, vascular endothelial growth factor (VEGF), basic fibroblast growth factor (bFGF), and matrix metalloproteinase (MMP)-9 to promote cancer progression and bone metastasis. Tumor-associated neutrophils (TANs) are able to release CXCR4, VEGF and MMP9 to promote tumor bone metastasis. Tumor cells also release factors such as RANK, E-cadherin, CXCR4, and parathyroid hormone-related protein (PTHrP) that promote osteolytic bone lesions.

In mineralized bone marrow, multiple cell types release signaling molecules that together make the bone microenvironment an attractive site for metastatic cancer cells to home. A “vicious cycle” develops that promotes metastasis to the bone. Osteoblasts and/or osteoclasts release various growth factors in the bone microenvironment, which further promote metastatic tumor growth and cause incurable osteoblastic and osteolytic lesions [2]. Early studies focused on the interactions between cancer cells and bone progenitor cells during bone metastasis. The significance of the contribution of the immune system in this process remains largely unexplored. Likewise, in vivo models that recapitulate the cancer cell-bone microenvironment interaction are lacking. It is most commonly accepted that the immune system functions as a major defense against cancer cells. However, increasing evidence suggests that metastasis may be dependent on the specific factors in the tumor microenvironment [3]. For example, an antitumoral or protumoral effect of the immune microenvironment may depend on the presence of accessory stromal cells, the local cytokine milieu, tumor-specific interactions and the specific types of immune cells present. As represented in Figure 1, for instance, cytotoxic T cells and natural killer cells indeed function as mediators of tumor clearance. Conversely, many other subtypes of immune cells including regulatory T cells (Tregs), CD4^+^ helper T cells, suppressive dendritic cells, and myeloid-derived suppressor cells (MDSCs) traffic to the bone-tumor microenvironment and are more prone to promote tumor progression and metastasis [4]. Likewise, as a response to the immune-suppressive cytokines secreted by tumor cells, the M1 macrophages and N1 neutrophils are subverted to tumor-associated M2 macrophages and N2 neutrophils which are characterized as having potent tumor-promoting activity [5]. In the current review, the detailed functions of different immune cells and their impact on cancer cell metastasis to the bone will be discussed. Additionally, the development of current therapeutic strategies for bone metastasis will be described.

## 2. Crosstalk among Cancer Cell, Immune Cells and the Bone Microenvironment

### 2.1. Bone Microenvironment

In multiple types of human cancer, the bone is the third most common site for metastasis [6]. The bone microenvironment plays a critical role in the development of metastases. In 1889, Stephen Paget proposed the “seed and soil” hypothesis: the dissemination of cancer cells (seed) from primary sites invades the metastatic sites (soil) to form metastatic lesions [7,8]. This hypothesis highlights that the bone microenvironment is fertile soil for metastasis, primarily due to: (1) high blood flow in the red marrow in bone; (2) tumor cell-stromal cell interactions; (3) multiple cells in the bone marrow that produce growth factors, angiogenic factors and bone-resorbing factors that stimulate tumor growth.

In order to understand the role of the bone microenvironment in tumor metastasis, the structure and function of bone and the cells that constitute the bone microenvironment are important to consider. As the connective tissue of the human body, bone provides structural support, protective function, and regulation of calcium levels [9]. Bones can be classified as long bones and flat bones, both of which contain 95% type-I collagen, 5% proteoglycans and other non-collagenous proteins [10]. Two major types of cells located within the bone microenvironment contribute to the metastatic bone niche: osteoblasts and osteoclasts. Osteoblasts develop from pluripotent mesenchymal stem cells in the bone marrow stroma that can transform to osteocytes which synthesize new bone matrix. Osteoblastic activity in bone metastases is primarily found in patients with prostate cancer [11]. In osteoblastic metastases, osteoblasts produce many factors including insulin-like growth factor 1 (IGF-1), insulin-like growth factor 2 (IGF-2), TGF-β, TNF-α and IL-1β that act as chemoattractants for cancer cells [12]. Meanwhile, osteoblasts interact with tumor cells to activate the Wnt signaling pathway and consequently increases bone formation which leads to osteoblastic lesions [13,14]. Osteoclasts are derived from monocytes and are responsible for bone resorption in the bone marrow [10]. Osteolytic lesions are more common than osteoblastic-type lesions in breast cancer, lung cancer, and especially renal cell cancer (RCC) [15]. The formation of osteolytic lesions is mediated by osteoclasts via the release of multiple osteoclastogenic factors from tumor cells (e.g., IL-1, IL-6, IL-11, PDGF, MIP1α, TNF, M-CFS, RANKL and PTHrP) [16,17]. These growth factors and cytokines are capable of stimulating metastatic growth in the bone, thereby establishing the vicious cycle of subsequent tumor adhesion and proliferation as well as further bone destruction [18]. In addition to immune cells, the bone microenvironment also contains stromal cells such as fibroblasts, adipocytes, vascular endothelial cells, chondrocytes, and osteoblasts, as well as transient cells such as erythrocytes, immune cells, and platelets that may play a role in metastasis. [10,19,20]. The non-cellular components of the bone, such as growth factors, cell adhesion molecules, cytokines, chemokines and calcium ions are released by multiple cell types including tumor cells in the bone marrow and also contribute to making the skeleton an attractive soil for metastatic cancer cells.

### 2.2. Cancer Cells Metastasize to Bone

For cancer cells to migrate from the primary tumor to the bone to initiate metastasis, disseminated tumor cells (DTCs) must escape from immune surveillance in order to survive in the circulation, and then extravasate into foreign tissues by attaching and adapting to the microenvironment at the metastatic site. For survival in the circulation, DTCs are covered by platelets in the bloodstream allowing them to evade the immune system and escape perforin/granzyme-mediated NK cell cytotoxicity and TNF-α-mediated cell death [21,22]. DTCs can acquire resistance to apoptosis by expressing prosurvival proteins such as BCL-2, MCL-1 and survivin-C that also protect DTCs against NK cell- or cytotoxic T cell-mediated killing [23]. Additionally, a common immune escape strategy includes the loss of MHC class-I molecules and the expression of programmed death ligand 1 (PD-L1) on tumor cells [24,25].

DTCs are attracted to the “nutrients” from fertile bone environments that allow tumor cells to colonize. In bone marrow, DTCs bind with osteoblasts through CXCR4/CXCL12 and annexin II/annexin II receptor interactions [26,27,28]. Furthermore, DTCs expressing E-cadherin on their surface can form adherin junctions with N-cadherin expressed on the surface of osteoblasts [29,30]. In addition, DTCs expressing RANK can interact with RANKL which is secreted by osteoblasts and osteocytes in bone environments [31,32]. DTCs also interact with multiple factors, such as VCAM-1, ICAM-1, vitronectin, osteopontin (OPN) and bone sialoprotein (BSP) via integrin receptors [19,33,34,35]. Once DTCs establish residency in the bone marrow, they can proliferate and compete with hematopoietic stem cells (HSC) for the endosteal niche, or enter a state of dormancy [36]. It is possible that dormant DTCs may never develop bone metastasis. Alternatively, they may exit the state of dormancy and form bone metastasis many years after initial diagnosis and treatment [37]. Despite the clinical significance of dormancy on tumor metastasis, the mechanisms underlying it remain poorly understood. Some evidence suggests that factors such as CXCL12, E-selectin, thrombospondin-1 (TSP1), Notch-1, bone morphogenetic protein (BMP) and TGFβ2 are involved in the regulation and maintenance of dormancy [38,39,40,41]. Once cancer cells exit the latency period of dormancy, they start to re-proliferate and form macrometastases. Evidence suggests that the release of stem cell signals by osteoclasts may trigger the NF-κB pathway to cause cancer cell reactivation [42]. Moreover, the release of Ca^2+^ during normal bone remodeling binds with CaSR, a calcium-sensing receptor to stimulate parathyroid hormone-related protein (PTHrP), that leads to tumor cell reactivation [43]. TGF-β1 secreted from neovascular tips and periostin secreted from cancer stem cells (CSCs) have also been associated with tumor cell reactivation in the bone microenvironment [38]. Furthermore, the activation of TGF-β, BMP, IGFs and PDGF family members released from the bone matrix has also been shown to stimulate tumor cell proliferation [44,45]. Once macrometastases are established, multiple factors such as M-CSF, TNF-α, IL-8 and IL-11 released from tumor cells drive osteoclasts to induce osteolytic lesions via the stimulation of RANKL [6,46].

### 2.3. Interaction between Immune Cells and Bone Microenvironment

Immune cells in the bone marrow are in close proximity to and associate with osteoclasts and osteoblasts in the bone microenvironment. For instance, immune cells interact with osteoclasts mainly through osteoprotegerin (OPG) /RANKL/RANK. CD4^+^ T cells can release factors such as IL-6, IL-11, IL-15, and TNF-α to enhance osteoclastogenesis and the formation of osteolytic lesions [47,48]. Activated CD4^+^ T cells can enhance OPG-L-mediated osteoclast activity and bone destruction [49]. The knockdown of RANKL in tumor-specific T cells reduced bone destruction and metastasis. Conversely, there is also evidence suggesting that the production of an inflammatory factor, interferon-γ (IFN-γ), by activated CD4^+^ T cells can inhibit the activity of osteoclasts [50,51,52]. In addition to the activation of T-cells, T cells can also establish a feedback loop that is regulated by osteoclasts. For example, osteoclasts secrete chemokines that recruit CD8^+^ T cells [53]. Taken together, this suggests that the balance between pro-tumorigenic and anti-tumorigenic effects of the immune cells in the bone microenvironment will determine the likely hood of bone metastasis.

Unlike osteoclasts, osteoblasts play a role in the regulation and the differentiation of all stages of B cell development [54]. Both B cells and macrophages have been shown to interact with osteoblasts. Additionally, macrophages can regulate osteoblast differentiation and mineralization both in vitro and in vivo [55]. The results from a recent study showed that CD169^+^ macrophages within the bone-tumor microenvironment were essential for tumor-induced bone formation by osteoblasts [56]. Hence, the osteoblastic niches should be considered a therapeutic target to prevent bone metastasis in certain tumors. In general, the mutual interaction between immune cells, osteoclasts and osteoblasts in the bone marrow microenvironment complicate the study and identification of the mechanisms that drive bone metastasis.

## 3. The Role of Immune Cells in Bone Metastasis

### 3.1. T Cells

Bone marrow is vascularized and represents a major part of the lymphocyte recirculation network, with billions of lymphocytes recirculating through it per day. The bone marrow microenvironment can provide lymphocytes with the appropriate support to develop, even in the absence of the thymus [57]. Approximately 8%–20% of mononuclear cells in the bone marrow are T cells or B cells with a ratio of 5:1 [58,59]. Among T cells, there are about 1.5% CD4^+^ T cells and 2.0%–2.5% CD8^+^ T cells. About one-third of CD4^+^ T cells are CD4^+^CD25^+^ regulatory T (Treg) cells [60,61,62]. Among T cells, the CD8^+^ cytotoxic T cell is one of the most important immune-mediated cells for tumor destruction. In the human body, T cells cannot recognize host proteins due to the process of immune tolerance to self-antigens. However, they can recognize the tumor-antigen-MHC-I-complex in the presence of antigen presenting cells (APCs), and thereby destroy tumor cells through the perforin-granzyme B- and/or Fas-Fas ligand axis-mediated apoptosis [3]. Unfortunately, the T cell-mediated anti-tumor immune responses are inhibited by TGF-β released from osteoclasts [63]. Thus, inhibitors of TGF-β may be effective enhancers of antitumor immune responses and ultimately prevent bone metastasis.

Among CD4^+^ T cells, Tregs are known to be potent immune suppressors that play important roles in maintaining homeostasis in the immune system. An increased number of activated Tregs have been observed in nearly all cancer patients, and dampen the immune response against cancer cells [64]. More importantly, the presence of Tregs in cancer patients predicts poor prognosis [65,66]. Maj et al. found that bone marrow Treg cells are significantly increased in patients with prostate cancer that has metastasized to the bone [67]. Zou et al. demonstrated that CXCR4/CXCL12 signaling mediates Treg cell trafficking to the bone marrow [68]. The results from those studies suggested that bone marrow is a preferential site for migration, selective retainment and function of Treg cells [68,69,70]. In addition to the immunosuppressive functions, FOXP3^+^ Tregs have been demonstrated as a major source of RANKL [71], the critical cytokine required for osteoclasts differentiation as well as cancer cell mobility and bone metastasis [72], suggesting RANKL^+^ Tregs may promote DTC recruitment to the bone.

CD4^+^ helper T cells (Th17 cells) are another important subset of CD4^+^ T cells that might be important in enhancing osteoclastogenesis and bone metastasis through the RANKL pathway [73,74]. Evidence suggests that tumor-specific Th17 cells enhance the activation of osteoclasts and induce osteolytic bone disease by producing RANKL [75]. Moreover, RANKL^+^ Th17 cell adoptive transfer into mice, orthotopically injected with 4T1 breast cancer cells, increases tumor-cell to bone colonization [75]. Interestingly, Th17 cells can differentiate into Treg cells during an immune response and in the presence of TGF-β1, aryl hydrocarbon receptor (AhR) activation promotes this conversion [76]. These results highlight the critical role and promising therapeutic potential of targeting Tregs and Th17 to prevent bone metastasis.

### 3.2. NK Cells

Apart from the cytotoxic T cell, another important cell-type in immune-mediated tumor killing is natural killer (NK) cells which belong to the innate immune system. There are approximately 0.4%–4% NK cells in the bone marrow [77,78]. Generally, NK cells do not recognize tumor-specific antigens, whereas, they recognize cancer cells directly through antigen-specific receptors such as NKG2D, CD16, DNAM1 and NCRs, which recognize ligands expressed on the surface of cancer cells [3]. Another way for NK cells to recognize cancer cells is through the recognition by “missing-self”, which is caused by the down-regulation of MHC molecules on cancer cells causing them to evade T cell recognition. Once NK cells bind to cancer cells, apoptosis occurs through granule-mediated-exocytosis or Fas-Fas ligand interactions [79]. Depletion of NK cells causes uncontrolled tumor growth and metastasis [80,81]. The Core2 β-1,6-*N*-acetylglucosaminyltransferase (C2GnT) plays an important role in NK cell-mediated tumor immunity. In the bone microenvironment, cancer cells expressing C2GnT disrupts the ligand-receptor-mediated (NKR/NKR-L and TRAIL/DR4) immune response blocking the apoptosis of cancer cells [82]. In addition to C2GnT, the E3 ubiquitin ligase Casitas B cell lymphoma-b (Cbl-b) attenuates the anti-tumor activity of NK cells by enhancing the activity of TAM tyrosine kinase receptors (Tyro3, Axl or Mer) on NK cells [81]. More importantly, TAM receptor inhibitors largely reduced breast cancer metastasis in animal models [81]. Additionally, NK cell dysfunction had been reported in esophageal squamous cell cancer, gastric cancer as well as prostate cancer [83]. An imbalance in the activating and inhibitory cell surface receptors on NK cells such as NKG2D and CD161, low expression of the signal transducing ζ chain, and down-regulation of cytotoxic machinery caused by immunosuppressive cytokines such as IL-10 and TGF-β may explain the mechanisms behind NK cell dysfunction frequently observed in the tumor microenvironments [84]. Reactive oxygen species (ROS) produced by granulocytes, macrophages and tumor cells in tumor microenvironment also play a role in NK cell dysfunction [85]. Thus, the improvement of NK cell survival and activation will enhance tumor-specific targeting.

### 3.3. Macrophages

Once monocytes exit the bone marrow and peripheral blood, they can enter tissues and differentiate into macrophages. Macrophages are mononuclear myeloid lineage cells originally known for their protective role in eliminating undesired pathogens [86]. Under the influence of various cytokines, macrophages can polarize into two different types of populations: the proinflammatory M1 subtype and the anti-inflammatory M2 subtype macrophage. M1 macrophages are commonly regarded as tumor-suppressing immune cells that secrete proinflammatory cytokines such as IL-1, IL-6, IL-23, IFN-γ and IL-12 which activate cytotoxic T cells and NK cells to eliminate cancer cells [87,88,89]. Conversely, M2 macrophages are regarded as tumor-associated macrophages (TAMs). TAMs have been studied extensively in primary tumors, and have been considered as one of the most important regulators of tumor progression, angiogenesis, invasion, and metastasis [86,90,91]. Many clinical studies have demonstrated that high levels of TAMs correlate with poor prognosis in many cancer types [91,92,93]. Distinct from M1, TAMs secret high levels of cytokines including IL-10 and TGF-β which decreases the activation of CD4^+^ and CD8^+^ T cells [94]. Likewise, emerging evidence including clinical data and animal experiments demonstrate that TAMs potentiate tumor metastasis [95,96,97], particularly bone metastasis [98]. Increased numbers of CD206^+^ M2-like macrophages have been found in prostate cancer with bone metastatic lesions [98,99]. Depleting macrophages via gene targeted or pharmacologic approaches inhibits tumor growth in bone in animal models [99]. Moreover, CD68 (phagocytic capacity marker) positive macrophages are increased in metastatic breast and prostate cancers compared to matched primary tumors [100,101]. Research into the potential mechanism of the role of TAMs in promoting bone metastasis has uncovered that chemokine (C-C motif) ligand 2 (CCL2)-expressing breast tumor cells engage CCR2^+^ stromal cells of monocytic origin, including macrophages and preosteoclasts, to facilitate colonization in lung and bone [102]. Additionally, CSF-1, which is a potent chemokine for regulating proliferation and differentiation of osteoclasts, monocytes and macrophages, has also been implicated in the contribution of macrophage-driven bone metastasis [103]. However, therapeutic targeting of CSF-1R restricts the recruitment of TAMs to primary sites in the MMTV-PyMT breast cancer model [104] and reduces osteolytic bone lesions in nude mice injected intracardially with MDA-MB-231 breast cancer cells [105]. Given the important role of macrophages in supporting cancer cell metastasis to bone, targeting macrophages will be an effective therapeutic treatment for bone metastasis.

### 3.4. Dendritic Cells

Dendritic cells (DCs), also known as professional antigen-presenting cells (APC), play a key role in the regulation of cytotoxic T-cell immune response activation by virtue of their antigen-presenting capacities. Many studies have demonstrated that tumor antigen-pulsed DCs are capable of inducing activation and proliferation of both T-helper cells and cytotoxic T cells to mediate anti-tumor immune responses [106,107]. A number of studies have evaluated the therapeutic potential of DC-based cancer vaccines for some tumors such as breast, lung, colon and prostate cancers [108]. Compared with spleen, liver or lung tissues, circulating DCs are prone to migrate to the bone marrow where microvessels constitutively express VCAM-1 and endothelial selectins which helps to retain DCs in the bone marrow microenvironment [109]. Although DCs are known for their powerful role in anti-tumor immune response, cancer cells may still influence DCs to promote an immunosuppressive phenotype.

In 2012, Sawant et al. found increased amounts of plasmacytoid DCs (pDCs) within the bone marrow of mice inoculated with 4T1 breast cancer cells [47]. PDCA-1 mediated deficiency of pDCs significantly reduced lung and bone metastasis [47], suggesting the potential for therapeutically targeting pDCs for the treatment of bone metastasis. Evidence also demonstrated that purified DCs from patients with breast cancer showed a significantly decreased ability to stimulate allogeneic T cells [110]. It has also been found that tumor-infiltrating DCs could suppress the cytotoxic capacity of CD8^+^ T cells via production of TGF-β, nitric oxide (NO), IL-10, VEGF and arginase I [5]. Furthermore, the recruitment of other immunosuppressive immune cells including Tregs and myeloid-derived suppressor cells (MDSCs) by pDCs could promote, rather than protect from, tumor progression and metastasis [111].

### 3.5. Myeloid-Derived Suppressor Cells

Myeloid-derived suppressor cells (MDSCs) are a heterogeneous population of cells comprised of immature myeloid cells that are generated in the bone marrow. Under normal conditions, the immature myeloid cells (IMCs) can differentiate into mature myeloid cells such as macrophages, dendritic cells and granulocytes. However, under pathological conditions including cancer, IMC differentiation is inhibited resulting in the accumulation and activation of immunosuppressive MDSCs by the production of immune suppressive factors such as arginase I, inducible nitric oxidase synthase and TGF-β from the IMCs [112]. By suppressing both innate and adaptive immune response, MDSCs assist in cancer progression and metastasis. Studies in pre-clinical animal models and human patients have demonstrated that MDSCs accumulate in almost all cancers, both in primary and metastatic solid tumors [89]. In the tumor microenvironment, MDSCs suppress T cell function by suppressing proliferation and promoting apoptosis of T cells. In addition to suppressing effector T cell populations, MDSCs can promote the expansion and activation of Tregs and in turn regulate immunosuppression. Also, MDSCs can secrete factors to promote angiogenesis and lymphangiogenesis which favor tumor growth. Meanwhile, pro-angiogenic growth factors produced by MDSCs can directly incorporate into the tumor endothelium [112,113]. Thus, many different mechanisms used by MDSCs allow cancer cell proliferation and metastasis to distant organs including the bone [114]. Zhuang et al. found that MDSCs can differentiate into osteoclasts and contribute to bone destruction in myelomas [115]. Sawant et al. reported that increased numbers of MDSCs in breast cancer could also drive bone metastasis during breast cancer progression in animal models [116]. During cancer cell colonization of bone, dysregulation of this process leads to increased osteoclast activation and osteolysis [116]. It should be noted that only MDSCs isolated from a bone microenvironment with bone metastasis were able to differentiate into mature and functional osteoclasts; whereas, MDSCs isolated from a tumor-bearing mouse without bone metastasis did not differentiate into active osteoclasts. This suggests that cancer cells resident in the bone microenvironment contribute to an increased number of activated osteoclasts. Given the potent effects of MDSCs on suppressing host immunity and promoting bone damage, bone marrow MDSCs may serve as a potential therapeutic target for bone metastasis.

### 3.6. Neutrophils

Neutrophils are an important component of the innate immune system and play key roles in the initiation, modulation, and resolution of the host immune response [117]. In the normal adult human body, neutrophils are generated at a rate of 1 to 2 × 10^11^ cells per day under normal conditions [118]. The bone marrow is a large pool for mature neutrophils and plays an important role in neutrophil homeostasis. The CXCR4/CXCL12 signaling pathway is required to maintain neutrophils [119]. Early studies have reported that neutrophils attack tumor cells through antigen 1-dependent recognition [120]. However, increasing evidence has revealed that a group of tumor-associated neutrophils (TANs) have a pro-tumor effect rather than an anti-tumor effect [5]. More specifically, the function of neutrophils in cancer is dependent on their subtype: a tumor-inhibitory N1 phenotype or a tumor-promoting N2 phenotype. The N1 phenotype neutrophils have anti-tumor and anti-metastatic function; whereas N2 phenotype neutrophils promote tumor angiogenesis, tumor cell dissemination, and metastatic seeding in distant organs including the bone [121]. TGF-β plays an important role in determining the neutrophil phenotype, by shifting the balance from an antitumor (N1) phenotype toward a pro-tumor (N2) phenotype [122]. TANs are able to release CXCR4, VEGF and MMP9, all of which have been implicated in the metastatic process [123]. Liu et al. showed that host Toll-like receptor 3 (TLR3) promotes lung pre-metastatic niche formation via neutrophil recruitment which further predicted poor patient survival [124]. Additional studies demonstrated that the neutrophil-to-lymphocyte ratio in cancer patients could be considered a prognostic biomarker for predicting the overall survival rate of cancer patients [125,126], and may also be a useful predictor of bone metastasis [127].

## 4. Therapeutic Potential for Bone Metastasis by Modulating the Immune System

### 4.1. Targeting T Cells

The increased understanding of the role of the immune system in the bone–tumor microenvironment has been translated into the development of immune system-modulating therapies. For instance, to enhance the immune response against tumors, CD8^+^ T cells can be stimulated by vaccination or engineering the T cell receptor (TCR) or chimeric antigen receptor (CAR) [3]. The therapeutic application of regulatory T cells has been well demonstrated in the clinic. Evidence suggests that certain chemotherapy regimens such as cyclophosphamide, fludarabine and paclitaxel-based chemotherapy are able to reduce Tregs (CD4^+^CD25^high^ regulatory T cells) through Fas-mediated cell death [128,129,130]. Studies also show that chemotherapeutic efficacy is improved with the addition of anti-CD25 treatments by mediating Treg regulation. Daclizumab and basiliximab are antihuman CD25 mAbs approved for use in cancer treatment as well as the treatment of other diseases. In metastatic breast cancer patients, treatment with daclizumab durably reduced circulating CD25^high^FOXP3^+^ Tregs favoring the population of tumor-specific cytotoxic CD8^+^T cells (CTLs) after vaccination with cancer antigen peptides (hTERT/survivin) [131]. The anti-CTLA-4 antagonist mAb such as ipilimumab and tremelimumab are used to block Foxp3^+^CD4^+^CD25^high^ Treg suppressive function by binding to Tregs receptors. In addition to anti-CTLA-4 antagonists, Anti-PD-1 antibodies nivolumab have also been used to restrict the suppressive function of Tregs [132]. In addition, the use of sunitinib and sorafenib which targets vascular endothelial growth factor receptor 2 (VEGFR2) reduces the number of peripheral blood Tregs [133].

The Toll-like receptors (TLR) are expressed on human Tregs. Studies showed that agonist TLR signaling (PAM2CSK4, PAM3CSK4, FSL-1) reduces Treg suppressive function via mechanisms involving downregulation of the Cdk-inhibitor p27Kip1 and restoration of the PI3K-Akt pathway [134]. Additionally, blocking Treg trafficking into the tumor site can be achieved by blocking the CCR4/CCL22 axis. Thus, Bayry et al. identified small-molecule chemokine receptor antagonists or mAb that block CCL22-mediated recruitment of human Tregs and Th2 cells in in vitro experiments [135].

### 4.2. Targeting NK Cells

NK cells have been recognized as promising agents for cell-based cancer therapies. Several clinical studies have been performed with adoptive autologous NK cells and allogeneic NK cell products in an attempt to target breast cancer, lung cancer, lymphoma, glioma, renal cell carcinoma, adenocarcinoma, leukemia, colorectal cancer, hepatocellular cancer, and melanoma [83]. There are other NK cell-based anti-cancer strategies such as genetic modification of NK cells. In this strategy, NK cells are modified to produce cytokines such as IL-2 and IL-15 which increase their survival capacity and proliferation and promote anti-tumor activity in vivo [136,137]. Similarly, to enhance their specificity for the target cells, NK cells can be modified to recognize antigens specifically expressed on the surface of cancer cells [83]. The combination of drug therapy with NK cell stimulating cytokines (IL-2, IL-12, IL-15, and IL-21) or immunomodulatory drugs (IMiDs) can enhance NK cell-mediated tumor killing [138]. Likewise, NK cell infusion may synergize with chemotherapy to enhance tumor targeting and elimination [139].

### 4.3. Targeting Macrophages

Many preclinical strategies targeting macrophages for suppression of the primary tumor as well as for metastasis are now being evaluated in the clinic, and provide proof of concept that targeting macrophages may enhance current anticancer therapies. Current strategies for targeting TAMs can be characterized into three main categories: depletion, reprogramming and molecular targeting. Given that bone marrow-derived macrophages (BDMs) are recruited to the tumor by chemokine (C–C motif) ligand 2/CCL receptor 2 (CCL2/CCLR2) or colony-stimulating factor-1 (CSF-1)/CSF-1R axis, inhibitors targeting these ligands and receptors have been developed [140]. Preclinical studies showed that blocking the CCL2/CCLR2 axis can suppress the accumulation of TAMs in tumors as well as reduce metastasis in animal models [141,142]. Discontinuation of CCL2 therapy accelerates breast cancer metastasis by promoting tumor angiogenesis [143]. Similarly, targeting CSF-1/CSF-1R signaling by small molecule pexidartinib (PLX3397) and mAb therapy (emactuzumab, cabiralizumab and PD-0360324) have been tested clinically and shown to reduce the number of TAMs in solid tumors as well as prevent metastasis [140,144,145,146]. Several compounds such as trabectedin, clodronate, and zoledronic acid have been demonstrated to deplete macrophages by inducing apoptosis [147,148]. To reprogram the suppressive effects of TAMs, sunitinib, sorafenib and fenretinide [4-hydroxy(phenyl) retinamide] have been used to inhibit STAT3 or STAT6 in macrophages, thereby inhibiting IL-10 secretion or skewing macrophage polarization [149,150]. Moreover, the strategies that exist for converting TAMs to antitumor macrophages including CSF-1R agonists, CD40 antagonists toll-like receptor (TLR) inhibitors, VEGF inhibitors, phosphatidylinositol-4,5-bisphosphate 3-kinase-g (PI3Kg) inhibition, and Class-IIa histone deacetylase (HDAC) inhibition, have been shown to result in macrophage-mediated reduction in primary tumor burden and distant metastasis [148].

### 4.4. Targeting Myeloid-Derived Suppressor Cells

MDSCs exhibit immunosuppressive activity by blocking the proliferation and activity of both T and NK cells [151]. Thus, MDSCs are a promising target in anticancer therapy. Currently, there are a variety of different therapeutic strategies that have been developed to target MDSCs including the following: (1) anti-Gr-1 antibodies and peptibodies that target membrane proteins on MDSCs eliminate MDSCs in various murine tumor models [152]; (2) chemotherapeutic agents (5FU, paclitaxel, gemcitabine, cisplatin, docetaxel and lurbinectedin), phosphodiesterase 5 (PDE-5) inhibitors (sildenafil, tadalafil and vardenafil), vemurafenib as well as zoledronic acid cause MDSC apoptosis thus reducing circulating MDSCs in patients [153,154]; (3) mTOR inhibitors (rapamycin) or STAT3 inhibitors (AG490, CPA7, S3I-201, and stattic) deactivate MDSCs [155,156]; (4) all-trans-retinoic acid (ATRA) or vitamin D promote the MDSC differentiation s into mature, non-suppressive cells such as macrophages and DCs [157]; (5) the COX2 inhibitor (celecoxib), PDE-5 inhibitors and nonsteroidal anti-inflammatory drugs NSAID (nitroaspirin) inhibit the suppressive function of MDSCs [158]; (6) tyrosine kinase inhibitors (sunitinib and sorafenib) inhibit both hematopoiesis and MDSCs production [154,159]; (7) antagonists for chemokine receptors (CCR2, CXCR2 and CXCR4) or chemokines (CCL2, CXCL5 and CXCL12) inhibitors prevent the recruitment of MDSCs from the bone marrow into tumor microenvironment [154,160,161].

### 4.5. Targeting Dendritic Cells and Tumor-Associated Neutrophils

Given that immature and/or dysfunctional DCs with immunosuppressive effects tend to accumulate in the tumor microenvironment, the ability to convert dysfunctional DCs into functional DCs could be a potential approach to enhance therapeutic efficacy. Studies have demonstrated that two families of microtubule destabilizing agents (dolastatin 10 and ansamitocin P3) can switch DCs from immunosuppressive to immune activating by provoking phenotypic and functional DC maturation [162]. Furthermore, vaccine immunotherapy with DCs is reported to prevent tumor metastasis [163]. Additionally, the therapeutic combination of mature DCs with 5-FU has been reported to suppress tumor metastasis [163].

Since TANs also act as a protumoral immune cells in many human cancers, targeting TANs may be a potential anticancer treatment strategy. Clinical studies found that CXCR2 or IL-17 inhibition could reduce neutrophil migration into the tumor [164,165]. In addition, the combination of immunotherapy with sunitinib could increase antitumor efficacy by interfering with immunosuppressive neutrophils in renal cell carcinoma patients [166]. Furthermore, since infiltrating neutrophils are driven by TGF-β to acquire a polarized N2 pro-tumor phenotype. Blockage of TGF-β leads to a shift from N2 to N1 phenotype of neutrophils with subsequent acquisition of antitumor activity [122].

## 5. Other Drugs Inhibiting Bone Metastasis

As described above, RANKL, which is secreted by bone marrow stromal cells, osteocytes, and tumor cells, is an essential mediator of osteoclast activity and osteolytic lesions, and is important for stimulating metastatic growth in the bone. Denosumab is a human monoclonal antibody that binds to and neutralizes human RANKL, inhibiting osteoclast activity and osteoclast-mediated bone destruction [167]. Many studies have been shown that denosumab reduce the frequency of skeletal-related events (SREs, defined as pathological fractures, surgery or radiation to bone, or spinal cord compression) in patients with advanced solid tumors, and increase their quality-adjusted life years [168,169,170]. Therefore, osteoclast inhibition with denosumab has provided an improvement for the management of patients with solid tumors and bone metastasis. Additionally, TGF-β, which secreted by osteoclasts and tumor cells, is also an important driver of tumor growth and immune suppression. Thus, inhibitors of TGF-β may be effective enhancers of antitumor immune responses and ultimately prevent bone metastasis. Galunisertib, a small molecule inhibitor of TGFβ receptor I (TGFβRI) [171], has been shown to inhibition of TGFβ-mediated immune-suppression decreasing tumor growth and bone metastasis [172,173,174,175].

## 6. Conclusions

Bone metastasis is a frequent occurrence in cancer patients, particularly patients with breast and prostate cancer. The abundant and specific cellular and molecular niches (such as hypoxia and tumor-derived factors) in the bone microenvironment, including high levels of multiple immune cell types, may impact tumor-to-bone metastasis, as well as contribute to bone pathologies in patients with bone metastasis [176,177]. Therefore, understanding the immune regulatory mechanisms in the bone marrow microenvironment is important for the development of cancer therapies. There are many promising therapeutic options based on reprogramming immune cells in bone marrow in current pre-clinical and clinical trials that give hope for improved treatments and outcomes in patients with metastatic bone cancer (Table 1). Furthermore, the combination of immunotherapy and conventional chemotherapy may synergistically result in reduced tumor progression and metastasis, as well as prolonged survival for cancer patients. Enhancing our understanding of this field will be required to develop effective therapeutic strategies that are urgently needed.

## Figures and Tables

**Table 1:** The role of immune cells in cancer progression and metastasis, and their therapeutic strategies.

Immune Cells	Functions		Therapeutic Strategies
	Anti-Tumoral	Pro-Tumoral	
CD8^+^ T cells	Recognize and eliminate cancer cells	—	Enhance CD8^+^ T cells immune response by vaccination or engineering the T cell receptor or chimeric antigen receptor
Tregs	—	Suppress immune response of cytotoxic T cells and NK cells that promote cancer cells survival and metastasis	Suppression of Tregs by chemotherapy with/or anti-CD25 treatment, anti-CTLA-4 antagonist mAb, anti-PD-1 antibodies, tyrosine kinase inhibitors, PI3K-Akt inhibitors, toll-like receptors inhibitors, CCR4/CCL22 antagonists
NK cells	Recognize and eliminate cancer cells	—	Stimulation of NK cells by cytokines such as IL-2, IL-15 and IL-21, or immunomodulatory drugs (IMiDs), or genetic modification of NK cells
Macrophages	Killing of cancer cells directly; Antigen-presenting function	Immunosuppression functions: TAMs promote survival and metastasis of cancer cells and angiogenesis	Suppress the accumulation of TAMs by CSF-1 inhibitors, CCL2 inhibitors; Depletion of TAMs by trabectedin, clodronate, and zoledronic acid; Reprogramming of TAMs by CD40 antagonism, toll-like receptor inhibitors, VEGF inhibitors, PI3Kg and HDAC inhibition, tyrosine kinase inhibitors, and fenretinide
Dendritic cells	Antigen presentation to T cells to stimulate immune response	Suppress the cytotoxic capacity of T cells and recruit immunosuppressive immune cells to promote tumor progression and metastasis	Targeting of DCs by microtubule destabilizing agents (dolastatin 10 and ansamitocin P3), chemotherapy, and vaccine immunotherapy
MDSCs	—	Suppress T cell functions; Regulate the immunosuppression; Stimulate angiogenesis and lymphangiogenesis	Targeting of MDSCs by chemotherapy, PDE-5 inhibitors, mTOR inhibitors, STAT3 inhibitors, COX2 inhibitors, tyrosine kinase inhibitors, chemokine receptors antagonists, peptibodies, vemurafenib, zoledronic acid, differentiation agents (ATRA), and vitamin D
Neutrophils	Attack cancer cells through antigen 1-dependent recognition	Release protumoral factors including CXCR4, VEGF and MMP9 to promote metastasis	Targeting of TANs by CXCR2 or IL-17 inhibition, or combination of immunotherapy with sunitinib
